# Meta-gene markers predict meningioma recurrence with high accuracy

**DOI:** 10.1038/s41598-020-74482-2

**Published:** 2020-10-22

**Authors:** Zsolt Zador, Alexander P. Landry, Benjamin Haibe-Kains, Michael D. Cusimano

**Affiliations:** 1 Division of Neurosurgery, Department of Surgery, St. Michael’s Hospital, Toronto, ON Canada; 2 Department of Computer Science, University of Toronto, Toronto, ON Canada; 3 Department of Medical Biophysics, University of Toronto, Toronto, ON Canada; 4 Princess Margaret Cancer Centre, University Health Network, Toronto, ON Canada; 5 Ontario Institute for Cancer Research, Toronto, ON Canada; 6 Vector Institute, Toronto, ON Canada

**Keywords:** Computational biology and bioinformatics, Genetics, Systems biology

## Abstract

Meningiomas, the most common adult brain tumors, recur in up to half of cases. This requires timely intervention and therefore accurate risk assessment of recurrence is essential. Our current practice relies heavily on histological grade and extent of surgical excision to predict meningioma recurrence. However, prediction accuracy can be as poor as 50% for low or intermediate grade tumors which constitute the majority of cases. Moreover, attempts to find molecular markers to predict their recurrence have been impeded by low or heterogenous genetic signal. We therefore sought to apply systems-biology approaches to transcriptomic data to better predict meningioma recurrence. We apply gene co-expression networks to a cohort of 252 adult patients from the publicly available genetic repository Gene Expression Omnibus. Resultant gene clusters (“modules”) were represented by the first principle component of their expression, and their ability to predict recurrence assessed with a logistic regression model. External validation was done using two independent samples: one merged microarray-based cohort with a total of 108 patients and one RNA-seq-based cohort with 145 patients, using the same modules. We used the bioinformatics database Enrichr to examine the gene ontology associations and driver transcription factors of each module. Using gene co-expression analysis, we were able predict tumor recurrence with high accuracy using a single module which mapped to cell cycle-related processes (AUC of 0.81 ± 0.09 and 0.77 ± 0.10 in external validation using microarray and RNA-seq data, respectively). This module remained predictive when controlling for WHO grade in all cohorts, and was associated with several cancer-associated transcription factors which may serve as novel therapeutic targets for patients with this disease. With the easy accessibility of gene panels in healthcare diagnostics, our results offer a basis for routine molecular testing in meningioma management and propose potential therapeutic targets for future research.

## Introduction

Meningiomas constitute approximately 34% of all brain tumors and affect approximately 3% of the adult population^1^, with an incidence rate of 8.36 and 3.61 per 100,000 person years in females and males, respectively^2^. For over half a century, prediction of meningioma recurrence has relied solely upon histological features^2^ (World Health Organization grading from I to III) and degree of surgical excision (Simpson grade). While about 70–80% of completely excised WHO grade I meningiomas do not recur, the remaining 20–30% do, with half of these recurrences occurring before the tenth year of follow-up^3^. For completely excised WHO grade II lesions, histology predicts a recurrence of 50% over 5 years and a disease-specific survival rate of 69% over 10 years. Given this lack of predictive accuracy, such patients require lifelong monitoring with MRI imaging and clinical follow-up. Not only does this represent a significant financial burden but the strain on the psychological well-bring of patients can be substantial given the associated ongoing uncertainty. It is therefore critical to develop more precise adjunctive methods of assessing recurrence risk that take into account the molecular biology of meningioma.

The genetic landscape of meningiomas is the next frontier to our understanding of its biology and its relation to disease recurrence, and many studies have begun to elucidate important molecular associations with aggressiveness and recurrence. For example, previous studies have identified chromosomal rearrangements^4^, mutations in genes TERT^5^, AKT1^6^, SMO^7^, DREAM complex repression^8^, and DNA methylation^9–11^ as correlates of tumor aggressiveness or recurrence, though translation into routine clinical practice remains limited to date. We add to the growing body of literature seeking to describe meningiomas in molecular terms by leveraging transcriptomics methods rooted in systems biology to capture small additive biological effects and relate them to complex and multifactorial clinical traits^12–14^. Such methods may help extract translatable markers of meningioma recurrence and avoid the potential stochasticity of using individual genes.

Gene co-expression networks are used to detect patterns in transcriptomic data by incorporating additive signal from relatively low gene expression levels^12,13,15^. This technique establishes gene similarity profiles based on shared connectivity profiles and clusters individual genes into co-expressed (biologically similar) modules. Expression of each module can be represented using principle component analysis to define a module meta-gene in order to reduce the noisy effects of individual genes. This approach seeks to holistically and robustly represent individual biological processes. Importantly, it is being increasingly used to identify novel disease-associated gene programs that would not be identified using single-gene approaches alone. Examples include the identification of gene programs underlying mouse weight^12^, glioblastoma^13^, and Huntington’s disease^14^. With the affordability of gene expression profiling, meta-gene-based prediction of tumor recurrence becomes feasible.

In the current study, we hypothesized the existence of gene modules that predict tumor recurrence with high accuracy. By annotating modules correlated with recurrent phenotypes we aim to provide further insight into the underlying biology driving tumor recurrence and identify potential new avenues for molecular therapeutics.

## Methods

### Data collection and pre-processing

We identified five datasets in the publicly available repository *Gene Expression Omnibus (GEO)*^16^ which contained human meningioma tissue transcriptomics and which were, at minimum, annotated with WHO grade and recurrence/progression. We define recurrence as the re-appearance of tumor on imaging after complete excision or the measurable progression of tumor after subtotal resection. Four of these studies use microarrays (GSE43290^17^, GSE16581^18^, GSE74385^19^, and GSE16181 (no citation available)) and one uses RNA-sequencing (GSE136661^8^) (Table 1). Gene expression analysis for these studies was carried out using microarray platforms Affymetrix Human Genome U133A, Affymetrix Human Genome U133 Plus 2.0, Illumina Human HT-12 V4.0, SU Homo Sapiens 912 (a custom-built chip with 912 cancer-related genes), and the high throughput sequencing platform Illumina HiSeq 4000, respectively. Raw data from each study was background corrected, log2 transformed and quantile normalized except GSE16181, which had previously been pre-processed similarly (the samples were background corrected, log2 transformed, and the signal intensity of each gene in a sample was divided by the 50^th^ percentile of all genes in that array). GSE16181 constituted the discovery cohort and external validation was done using a merged dataset from the remaining microarray-based series (validation cohort 1) which was batch-corrected with *ComBat*, a well-established Bayesian batch correction tool^20^, and the RNA-sequencing series GSE136661 (validation cohort 2).

**Table 1:** Study demographics.

GEO entry	N patients (n recurrence)	Mean age (SD)	N male (%)	Median F/U^1^ (range)	Median TTR^2^ (range)	WHO grade (I II III) [n]
GSE16181	252 (92)	44.1 (12.7)	53 (21.0)	10.0 (1.0–15.0)	3.0 (1.0–14.0)	140 88 24
GSE43290	47 (8)	61.7 (15.0)	13 (27.7)	3.5 (1.4–25.4)	N/A	33 12 2
GSE16581	16 (6)	58.3 (11.1)	7 (43.8)	4.4 (0.3–8.6)	N/A	8 7 1
GSE74385	45 (22)	N/A	N/A	N/A^3^	N/A	17 8 20
GSE136661	145 (22)	58.0 (13.5)	52 (35.8)	N/A^4^	N/A	116 29 0

^1^Follow-up (years).

^2^Time to recurrence (years).

^3^Follow up at least 3 years for non-recurrent tumours, though specific times are not published.

^4^Follow up reported as 0 to 91 months (up to 7.6 years) with a median of 28 months (2.3 years), though specific times are not available.

### Gene module detection and meta-gene computation

Our analysis was based on the well-established Weighted Gene Correlation Network Analysis (WGCNA), which has been described in detail elsewhere^13^. Modules were discovered using the discovery cohort. We first constructed an adjacency matrix with gene–gene Pearson correlations. Soft-thresholding was introduced by raising correlations to a common power, selected as the lowest natural number for which the network approached scale-free topology (*r*^*2*^ ≥ 0.9). The adjacency matrix was subsequently converted into a biologically-inspired topological overlap map (TOM), wherein pairwise gene similarities are computed based their shared connectivity profiles within the network, a more meaningful measure than direct correlation^21^. Hierarchical clustering was done on the TOM and clusters (gene modules) selected with hybrid adaptive tree cut^22^, an unsupervised dendrogram cutting function which considers not only the degree of correlation but also the shape of the dendrogram to determine clusters. Finally, representative module “meta-genes” for each sample are taken as the projections of a sample’s modules’ gene expression values on their respective first principal components. The validity of this approach is demonstrated in Supplemental Fig. 1.

**Figure 1:**
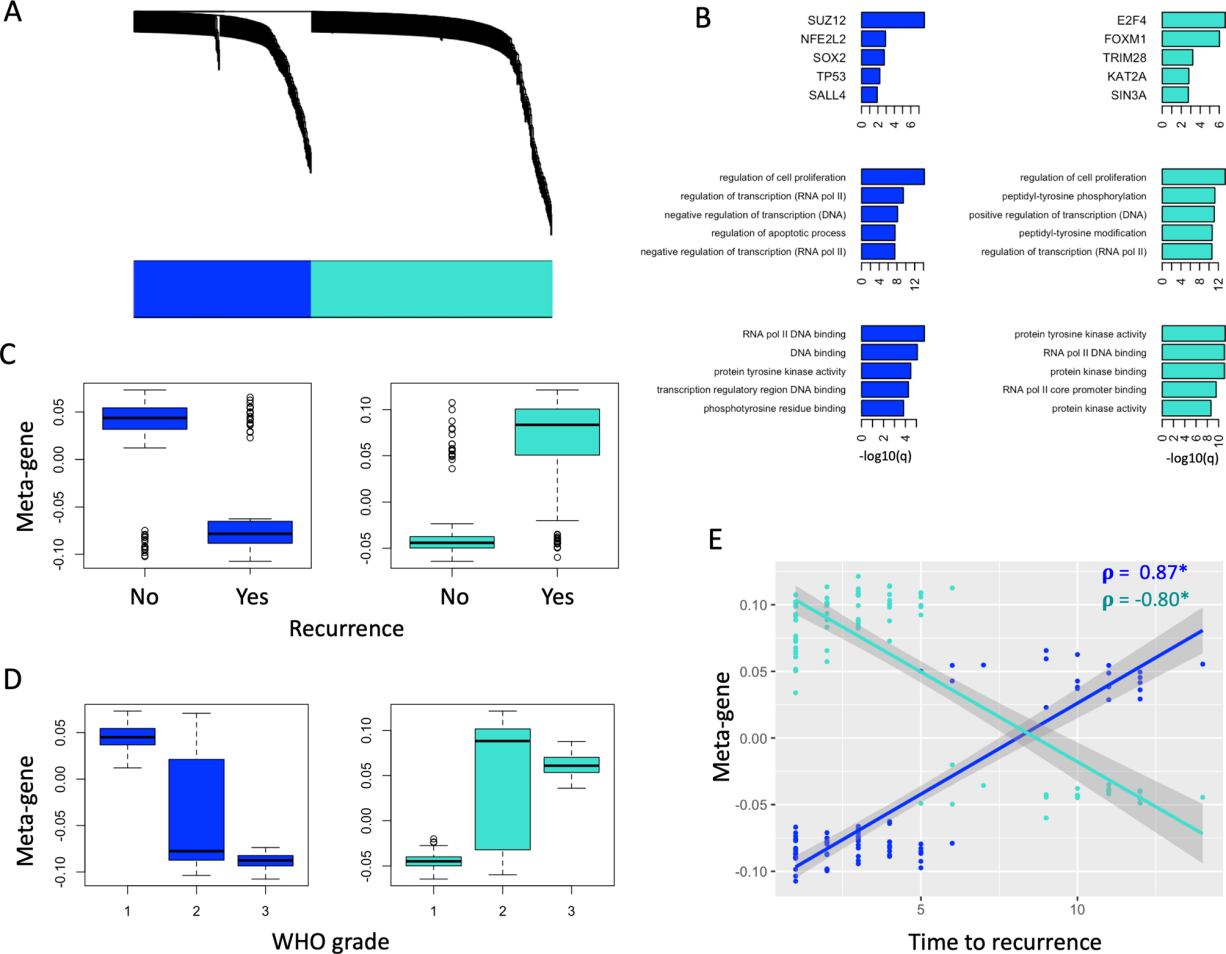
Gene module detection and characterization. (**A**) Weighted gene co-expression network dendrogram demonstrates hierarchical clustering of individual genes. Module annotation of individual genes is represented by the colour bar, which reveals 2 significant modules (blue and turquoise). (**B**) Module gene lists are annotated using Enrichr. The top 5 transcription factors (ENCORE/CHEA consensus TFs) and entries for GO Biological Processes and GO Molecular Functions are represented in the top, middle, and bottom rows, respectively. Ranking is by − log10(q-value). Color corresponds to the module colour from (**A**). (**C**,**D**) Boxplots comparing meta-gene expression between recurrent and non-recurrent tumors (**C**) and WHO grades (**D**) for both modules. (**E**) Correlation between time to recurrence (years) and meta-gene expression of both modules. Pearson correlation is represented by $\rho$. *p < 0.05.

### Module characterization

We first selected modules with significantly different meta-gene expression between recurrent and non-recurrent tumors (t-test *p* < 0.05) in the discovery cohort. In order to probe the biological function of relevant modules, we used the well-established and open-source *Enrichr*^23,24^ to analyze their constituent gene lists. Highly associated GO Biological Processes, GO Molecular Functions, and transcription factors (from the CHEA/ENCODE consensus list) are shown in Fig. 1.

### Recurrence classifier

**Figure 2:**
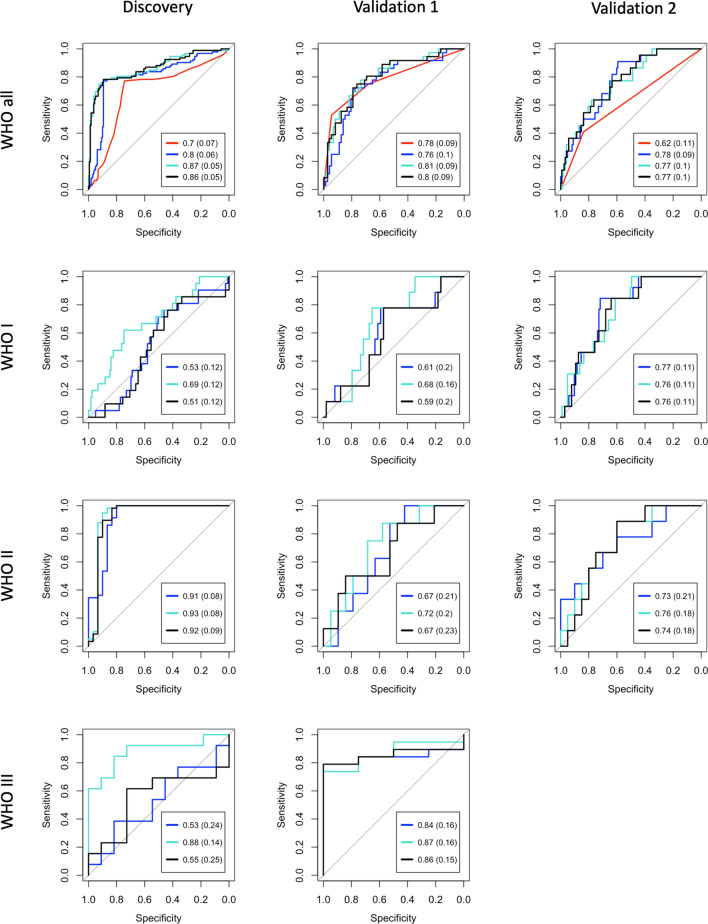
Logistic regression classifier performance. For each receiver operative characteristic curve, black represents a model containing both modules while blue and turquoise correspond to their respective individual modules (SUZ12-enriched and E2F4/FOXM1-enriched, respectively). The top row (including all WHO grades) includes a red curve which represents the predictive accuracy of WHO grade alone. Note that with the exception of the black curves, each model contains a single predictive variable (metagene or WHO grade). Models in rows 2–4 include only individual WHO grades as labeled. Columns (left to right) represent performance on the discovery cohort (tenfold cross validation), merged microarray validation cohort (Validation 1), and RNA-seq validation cohort (Validation 2). For the models including all WHO grades, the red curves are statistically less predictive (DeLong *p* < 0.05) than all other curves in the Discovery and Validation 2 cohorts, while all curves in Validation 1 are similar to one another (DeLong’s *p* < 0.05).

**Figure 3:**
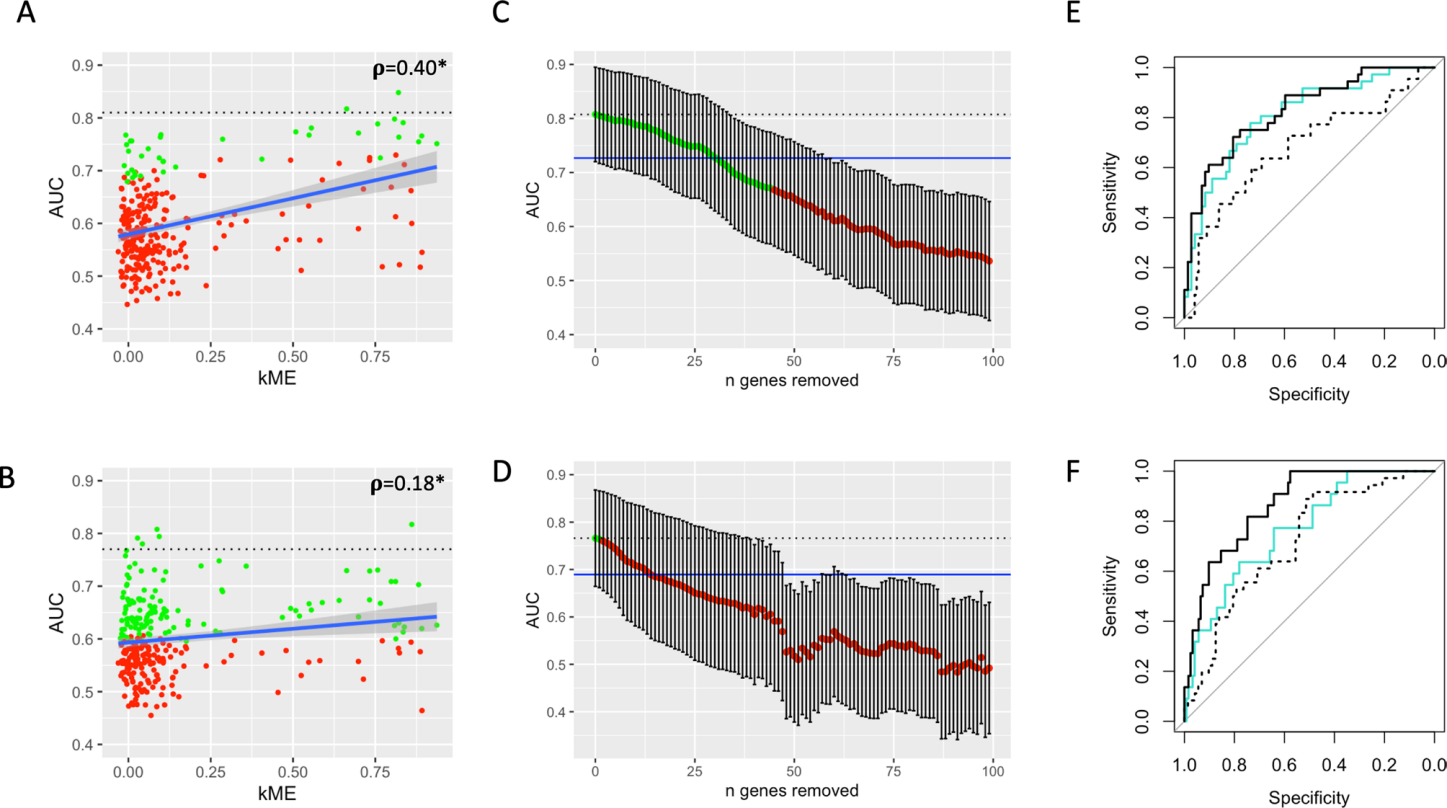
E2F4/FOXM1-enriched module performance. (**A**,**B**) Correlation between kME (the correlation of a gene’s expression to the E2F4/FOXM1-enriched module meta-gene) and the recurrence predictive accuracies (AUCs) of the module’s constituent genes as assessed in validation cohorts 1 (**A**) and 2 (**B**). The interrupted black line represents the module metagene performance. Green points represent genes that whose ROC curves are similar to the module metagene (DeLong’s *p* > 0.05), while red points represent genes which are significantly different (DeLong’s *p* < 0.05). (**C**,**D**) AUCs of ROC curves generated from iteratively removing genes from the module in descending order of highest AUC in validation cohorts 1 (**C**) and 2 (**D**) as computed in (**A**,**B**) (and iteratively re-calculating the resultant meta-genes). A maximum of 100 top genes are removed from each cohort. Dotted lines and dot colors are as in (**A**,**B**). Blue line corresponds to 90% of the absolute AUC of the full model. Thirty of the top genes in validation cohort 1 must be removed before the updated module metagene yields an AUC less than 90% of the full model (sparse model 1), and thirteen in validation cohort 2 (sparse model 2). Notably, only two genes overlap between these sparse models. (**E**) ROC curves comparing the full model in validation cohort 1 (solid turquoise curve) to sparse model 1 (meta-gene of the 30 genes extracted above) on validation cohorts 1 (solid black curve) and 2 (broken black curve). All curves are statistically similar (DeLong’s *p* > 0.05). (**F**) ROC curves comparing the full module in validation cohort 2 (solid turquoise curve) to sparse model 2 (meta-gene of the 13 genes extracted above) on validation cohorts 2 (solid black curve) and 1 (broken black curve). The AUC of the solid black curve is significantly higher than the other two (DeLong’s *p* < 0.05).

**Figure 4:**
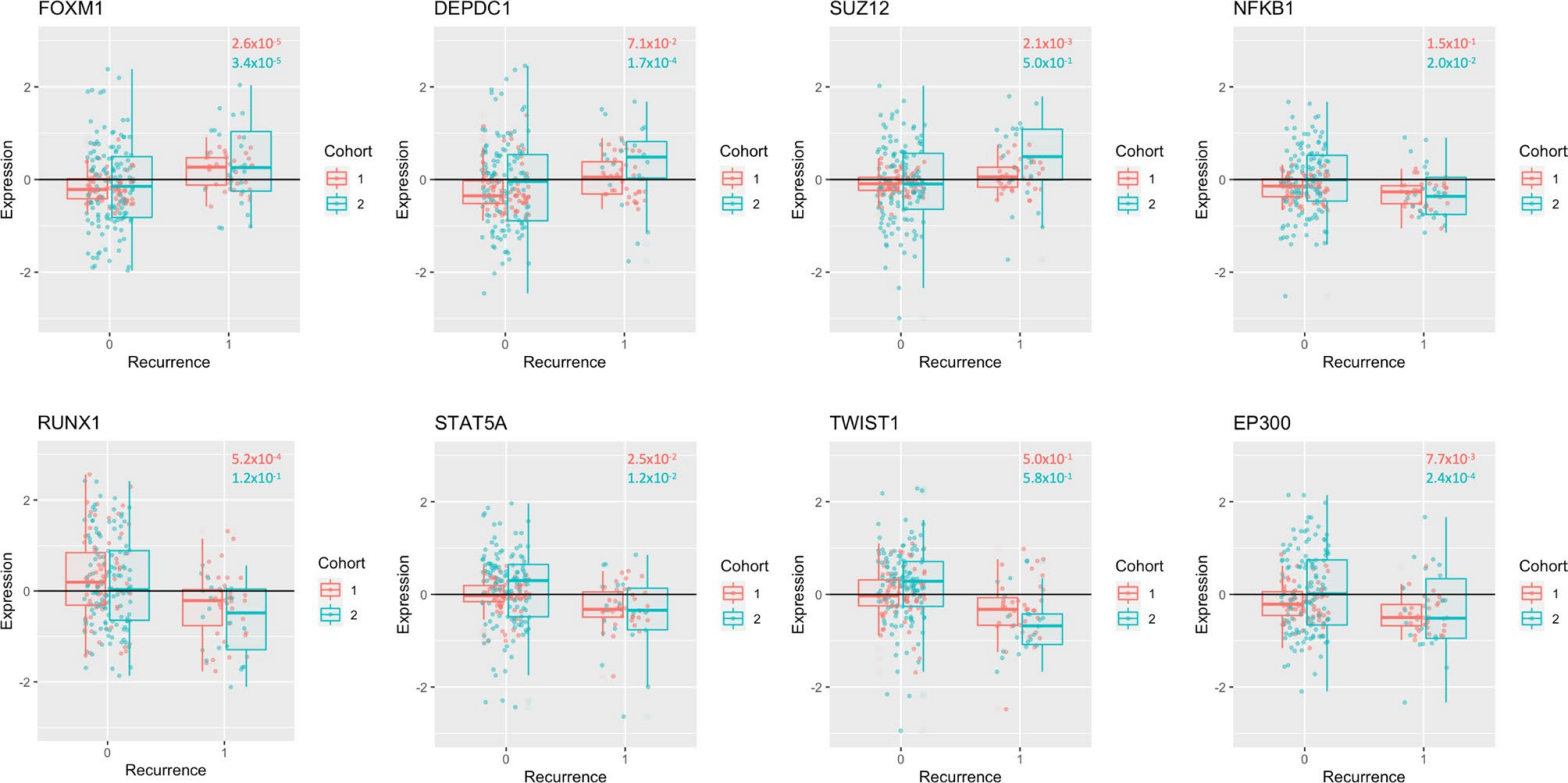
Transcription factors associated with the E2F4-enriched module. Transcription factors with Enrichr q-value < 0.05 and t-test *p* < 0.05 between recurrent and non-recurrent tumors in both validation cohorts are included. Red corresponds to validation cohort 1 (microarray) and blue corresponds to validation cohort 2 (RNA-sequencing). ANOVA *p*-values for gene association with WHO grade are found on the plot, with colour corresponding to boxplot colours. Note that DEPDC1 and NFKB1 are not significantly associated with WHO grade in validation cohort 1, SUZ12 and RUNX1 are not significantly associated with WHO grade in validation cohort 2, and that TWIST1 is not significantly associated with WHO grade in either validation cohort.

A logistic regression classifier was used as our prediction model, with module meta-genes as regressors and recurrence as a binary response (Fig. 2). The model was trained with the discovery cohort and tested with tenfold cross validation on the discovery cohort and separately with the two external validation sets. Performance was also tested by stratifying each model by WHO grades. Differences between receiver operating characteristic curves were assessed used DeLong’s *p*-value, as computed with the *pROC* package in R^25^. A multivariate logistic regression model was used to verify that modules provide prognostic information even when controlled for WHO grade in all cohorts and, given the depth of annotation in validation cohort 2, this model is also controlled for Simpson grade. Module performance was further assessed by computing the predictive accuracies of constituent individual genes and assessing the performance of sparsified models containing only the most individually predictive genes (Fig. 3, Supplemental Fig. 3). Finally, potential driver transcription factors (from Enrichr, q-values < 0.05 considered significant) were annotated based on their association with tumor recurrence (t-test < 0.05 considered significant) in both validation cohorts (Fig. 4).

### Computational platform

All analysis was performed using R, an open-source platform for statistical computation and graphics^26^.

### Ethical approval

This article relies entirely on openly available data from previous studies, and thus ethics approval is not required.

## Results

An overview of patient demographics can be found in Table 1. The discovery cohort (GSE16181) comprised 252 tumors, of which 92 (37%) recurred. Patients were distributed between WHO grade I (140; 56%), II (88; 35%), and III (24; 9%). Median follow up was 10 years (range 1–15) and median time to recurrence was 3 years (range 1–14). Validation cohort 1 (merged microarray) consisted of 108 tumors and 36 recurrences (33%). Tumors consisted of WHO grade I (58; 54%), II (27; 25%), and III (23; 21%). Finally, validation cohort 2 (GSE136661) consisted of 145 patients (22 recurrences; 15%). One hundred sixteen (80%) were WHO grade I and 29 were WHO grade II (20%). This cohort was also annotated with Simpson grade (53 grade 1 [37%], 60 grade 2 [41%], 31 grade 4 [21%], and 1 unknown [1%]), MIB (mean 6.4; SD 7.5), chromosome 22 loss (73 loss, 54 no loss, 18 unknown), and TERT promoter mutation (19 mutants, 84 wild type, 42 unknown).

Co-expression networks revealed two gene modules (Fig. 1) with significant association to recurrence (t-test *p* < 0.05) and WHO grade (ANOVA *p* < 0.05): a “blue” module containing 220 genes, and a “turquoise” module containing 299 genes (Supplemental data; Fig. 1C,D; Supplemental Figs. 2 and 4). We note that all but one input gene falls into one of the modules using adaptive tree cutting (Fig. 1A), which is likely explained by the design of the array (consisting of genes highly correlated with pro-cancer anti-cancer mechanisms). The meta-gene of each module was defined as the first principal component of its expression values^15^. Annotating module genes with Enrichr reveals transcription factors SUZ12, NFE2L2, SOX2, TP53, SALL4 to be strongly associated with the blue module (hereafter called the “SUZ12-enriched” module), and E2F4, FOXM1, TRIM28, KAT2A, SIN3A to be strongly associated with the turquoise module (hereafter called the “E2F4/FOXM1-enriched” module). Annotating with biological processes and molecular functions yield several mechanisms related to cell cycle and transcription (Fig. 1B). We note that the SUZ12-enriched module has a strong positive correlation with time to recurrence (Pearson correlation 0.87, *p* < 0.05) while the E2F4/FOXM1-enriched module has a strong negative correlation (Pearson correlation − 0.80, *p* < 0.05) (Fig. 1E). This further supports the E2F4/FOXM1 module as a marker of aggressiveness in meningioma.

We next investigated whether the meta–genes could be used to predict meningioma recurrence using a logistic regression model (Fig. 2). Notably, the E2F4/FOXM1-enriched module consistently achieved equal or greater performance (AUC) than the SUZ12-enriched module and both modules combined. We achieve good performance from this module in the discovery cohort (tenfold cross validation AUC = 0.87 ± 0.05) and both validation cohorts (AUC = 0.81 ± 0.09 and AUC = 0.77 ± 0.1 in validation cohorts 1 and 2, respectively). Notably, the performance of all module meta-gene predictors was better than WHO grade in the discovery and RNA-seq validation cohorts (DeLong *p* < 0.05). The E2F4/FOXM1-enriched module meta-gene remains a predictor of recurrence when correcting for WHO grade in all cohorts, and in the RNA-seq validation cohort it remains independently predictive when also correcting Simpson grade (Table 2). Notably, only one patient was excluded from the model which included Simpson grade due to missing data; the other three models included all samples. We also investigated the E2F4/FOXM1-enriched module classifier performance for individual WHO grades and achieved modest performance for all permutations. For WHO grade 1, AUCs are 0.69 ± 0.12 (discovery), 0.68 ± 0.16 (validation 1), and 0.76 ± 0.11 (validation 2). For WHO grade 2, AUCs are 0.93 ± 0.08 (discovery), 0.72 ± 0.2 (validation 1), and 0.76 ± 0.18 (validation 2). For WHO grade 3: 0.88 ± 0.14 (discovery) and 0.87 ± 0.16 (validation cohort 1). We suspect that relatively poor performance seen in the WHO grade 1-only cohort is related to the rarity of recurrence labels.

**Table 2:** Model performance by cohort.

Series	Predictors^1^	OR (95%CI)^2^	*p* value^2^
Discovery	M + WHO	666.73 (249.80–1779.55)	< 2 × 10^–16^
Validation 1	M + WHO	4.17 (1.63 – 10.69)	3.7 × 10^–3^
Validation 2	M + WHO	0.26 (0.12 – 0.55)	6.0 × 10^–4^
Validation 2	M + WHO + SG	0.24 (0.12 – 0.48)	7.5 × 10^–5^

^1^ M = Module, WHO = WHO grade, SG = Simpson Grade.

^2^Odds ratios and p-values refer to the module performance within the multivariate model.

Given the E2F4/FOXM1-enriched module performance, we sought to better understand it’s internal gene structure (Fig. 3; Supplemental Fig. 3). We find there is weak positive correlation between kME (the correlation between a gene’s expression and it’s representative meta-gene’s expression) and individual gene predictive accuracies (external validation AUC) for both validation cohorts (Fig. 3A,B). However, the data is quite noisy and thus demonstrates the considerable benefit of co-expression analysis. In order to determine module stability, we sequentially removed the most individually predictive genes and re-calculated the resultant meta-gene classifier performance until the module performance (AUC) dropped to 90% of it’s original AUC (Fig. 3C,D). Thirty genes were removed in validation cohort 1 and 13 in validation cohort 2; we define these top-performing genes as sparse modules 1 and 2, respectively. Notably, only 2 genes overlap in these sparse modules. The meta-gene computed from sparse module 1 performs similarly to the full E2F4/FOXM1-enriched module in validation cohort 1 (DeLong *p* > 0.05). It also performs similarly to both of these models when applied to validation cohort 2. The meta-gene from sparse module 2 performs significantly better than the E2F4/FOXM1-enriched module in validation cohort 2 (DeLong’s *p* < 0.05), though the performance of the E2F4/FOXM1-enriched module and sparse module 2 in validation cohort 1 are similar (Fig. 3E,F). Importantly, neither of the sparse models exhibit consistently improved performance over the full model (any gain in one validation cohort is lost in the other as shown in Supplemental Fig. 3). This analysis suggests that the redundancy of the larger models adds to its generalizability across different samples and data acquisition techniques.

We also sought to characterize transcription factors (TFs) associated with the E2F4/FOXM1-enriched module using the Enrichr data (Fig. 4). TFs significantly associated with module genes (q-value < 0.05) with significantly different expression between recurrent and non-recurrent tumors in both validation cohorts (t-test *p* < 0.05) are examined in Fig. 4. Notably, three TFs are upregulated in recurrent tumors (FOXM1, DEPDC1, SUZ12) and five are downregulated (NFKB1, RUNX1, STAT5A, TWIST1, and EP300). Importantly, TWIST1 is not significantly associated with WHO grade (ANOVA *p* > 0.05) in either of the validation cohorts, and DEPDC1, SUZ12, NFKB1, and RUNX1 is not significantly associated with WHO grade one of the validation cohorts.

Finally, we performed a gene linkage analysis on module genes to assess the importance of chromosomal gains/losses in meningioma biology (Supplemental Fig. 5). We note that the module genes are distributed throughout the genome and thus it is not possible to explain the biology we observe in terms of single large chromosomal gains/losses. However, we note a significant correlation between the degree of gene separation on a chromosome and their co-expression, which gives credence to the need for studying this biology in terms of gene programs rather than individual genes.

## Discussion

We have used a simple and robust approach to predict meningioma recurrence using gene modules derived from a list of cancer-associated genes, which yields high accuracy in two external validation cohort: one merged microarray-based cohort and one RNA-seq based cohort. Predictive ability of the E2F4/FOXM1-enriched module remained modest when controlling for WHO grade in all 3 cohorts. In the highly annotated validation cohort 2, this module remained predictive of recurrence when also controlling for Simpson grade. We also demonstrate the robustness of a module approach, showing that while predictive accuracies can be improved on an individual cohort by sparsifying the module into the its most individually predictive genes, any added performance is lost on other cohorts. This lends credence to the value of a gene program-based approach. Finally, we identify transcription factors which are strongly associated with the top-performing module and whose expression is significantly different between recurrent and non-recurrent tumors. In particular, FOXM1, DEPDC1, and SUZ12 are positively associated with recurrence while RUNX1, STAT5A, TWIST1, EP300 and NFKB1 are negatively associated.

These results lend to the value of this semi-supervised approach to genetic analysis and yields a panel of genes which could effectively be represented on an inexpensive mini-microarray chip for clinical use. Additionally, some of the associated transcription factors have previously been implicated in meningioma. For example, FOXM1 has been shown to be a critical driver of meningioma aggressiveness and is associated with the activating DREAM complex, thereby allowing cell-cycle progression and cell proliferation^8^. SUZ12 is known as an element in PRC2, which is involved in chromatin silencing and appears to be lost in a subset of comparatively indolent meningioma^8^. EP300, an apoptosis-associated transcription factor, has been shown to be downregulated in meningioma when compared to normal arachnoid^27^ though it’s role in meningioma prognostication is not well understood. While DEPDC1 has not been studied in meningioma, it is a known regulator of NFkB signaling and is overexpressed in multiple cancers such as glioma, breast cancer, and nasopharyngeal cancer, having been proposed as a potential therapeutic target in each^28–30^. Similarly, the roles of RUNX1, STAT5, and TWIST1 in meningioma remain poorly understood, and could represent novel therapeutic targets.

In our study, we demonstrate the potential utility of a meta-gene based systems biology approach in meningioma prognostication. This principle can be used in marker discovery for other challenging diseases where conventional approaches such as differential gene expression analysis have been unsuccessful. Our results are derived from data obtained under diverse conditions and yet still produce an accurate classier which projects translational value in creating a diagnostic panel. The additional mapping of potentially key transcription factors lends to the therapeutic potential of this holistic approach as well, though validation studies would be needed to support this hypothesis.

Our study uses publicly available data and is thus limited by sparse annotation and variable follow-up times. The discovery cohort is not associated with a peer-reviewed citation, and thus the full extent of their methodology is not available. The extent of tumor excision is not known in all cohorts. Although this is conventionally held to affect recurrence^31^ the relevance has been debated over the past decade and our classifier yields high accuracy purely with gene expression data. Additionally, in validation cohort 2, our classifier is predictive of recurrence independently of Simpson grade. While follow-up times are limited for some patients (Table 1) they still represent real life data from general neurosurgical practice. This does, however, limit our ability to perform further analysis such as Kaplan–Meier survival prediction and may affect results given that we show time dependence of the correlation between metagene expression and recurrence (Fig. 1). Additionally, we note that the behaviour of these metagenes appears to depend on the cohort being investigated (though always are prognostic of recurrence), which makes choosing a clinically relevant cut-off point impossible. Achieving clinical value would therefore require the use of a training cohort. Though our results are largely exploratory at present, further optimization of a predictive gene module using prospectively gathered cohorts with greater follow up times may ultimately translate into a novel, personalized approach to care for patients with meningioma.

## Conclusions

We apply gene co-expression networks to a microarray-based cohort of 252 adult meningiomas and identify a gene module which predicts tumor recurrence with high accuracy in both microarray and RNA-seq external validation cohorts. Importantly, the module remains predictive when controlling for WHO grade in all three cohorts. With the wide accessibility of custom-made mini-arrays, our findings support, and should encourage, the shift to include this or a similar classifier in routine clinical care. Furthermore, the module’s strong association with key transcription factors may yield new therapeutic options for patients with meningioma.

## Supplementary information

Supplementary Figures.

Supplementary Information.

## Data Availability

All data is available and was retrieved from the publicly accessible online genetic repository GEO omnibus (https://www.ncbi.nlm.nih.gov/geo/).
