# Supplementary material for: Meta-gene markers predict meningioma recurrence with high accuracy
Source: Sci Rep. 2020 Oct 22;10:18028. doi: 10.1038/s41598-020-74482-2 (PMC7582964; doi:10.1038/s41598-020-74482-2)
Supplement: Supplementary file 1 — Supplementary Figures. [file 41598_2020_74482_MOESM1_ESM.pdf]

**SUPPLEMENTAL FIGURE: Meta-gene markers predict meningioma recurrence with high accuracy independent of WHO grade**

Zsolt Zador*[1], Alexander P. Landry*[1], Benjamin Haibe-Kains[2,3,4,5,6], Michael D. Cusimano[1]

1) Division of Neurosurgery, Department of Surgery, St. Michael's Hospital, Toronto, ON, Canada

2) Department of Computer Science, University of Toronto, Toronto, ON, Canada;

3) Department of Medical Biophysics, University of Toronto, Toronto, ON, Canada;

4) Princess Margaret Cancer Centre, University Health Network, Toronto, ON, Canada;

5) Ontario Institute for Cancer Research, Toronto, ON, Canada;

6) Vector Institute, Toronto, ON, Canada;

**Co-Corresponding Authors:**

Zsolt Zador

Division of Neurosurgery, Department of Surgery, St. Michael's Hospital,

Email: zadzso@gmail.com

Alexander Landry

Division of Neurosurgery, Department of Surgery, St. Michael's Hospital

Email: alex.landry@mail.utoronto.ca

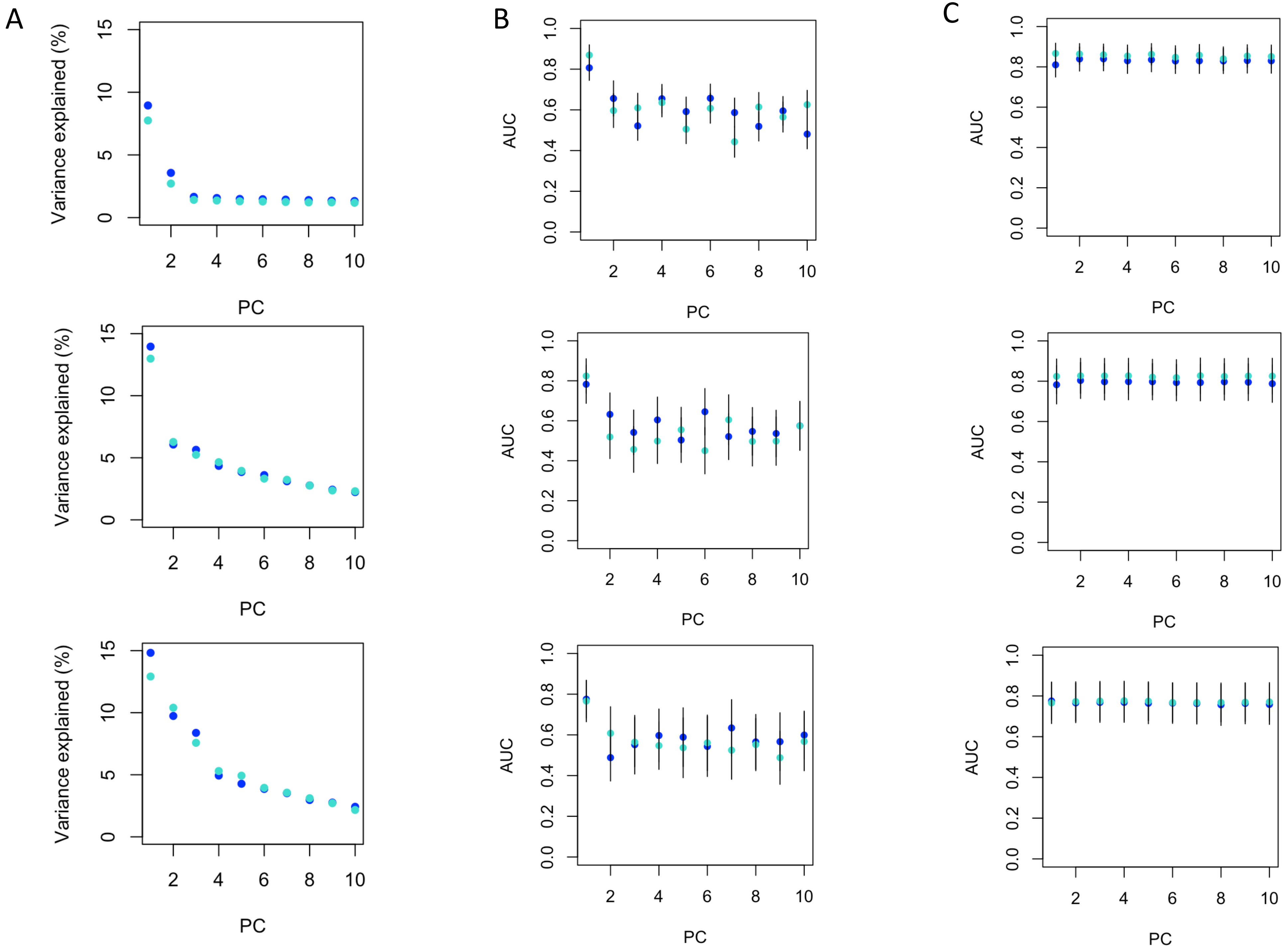
A
B
C
Variance explained (%)
AUC
PC

**Supplemental Figure 1:** Validation of using the first principle component as module meta-gene. Top row represents discovery cohort, middle row represents validation cohort 1 and bottom row represents validation cohort 2. A: Proportion of variance explained by the first 10 principle components of both modules. B: Predictive accuracy (AUC) using single principle components as metagenes in the classifier, demonstrating that the first principle component is the most informative (this is the definition of the module meta-gene). C: Accuracy achieved by sequentially adding principle components to the metagene computation in the GLM, demonstrating that adding higher principle components does not improve the classification accuracy.

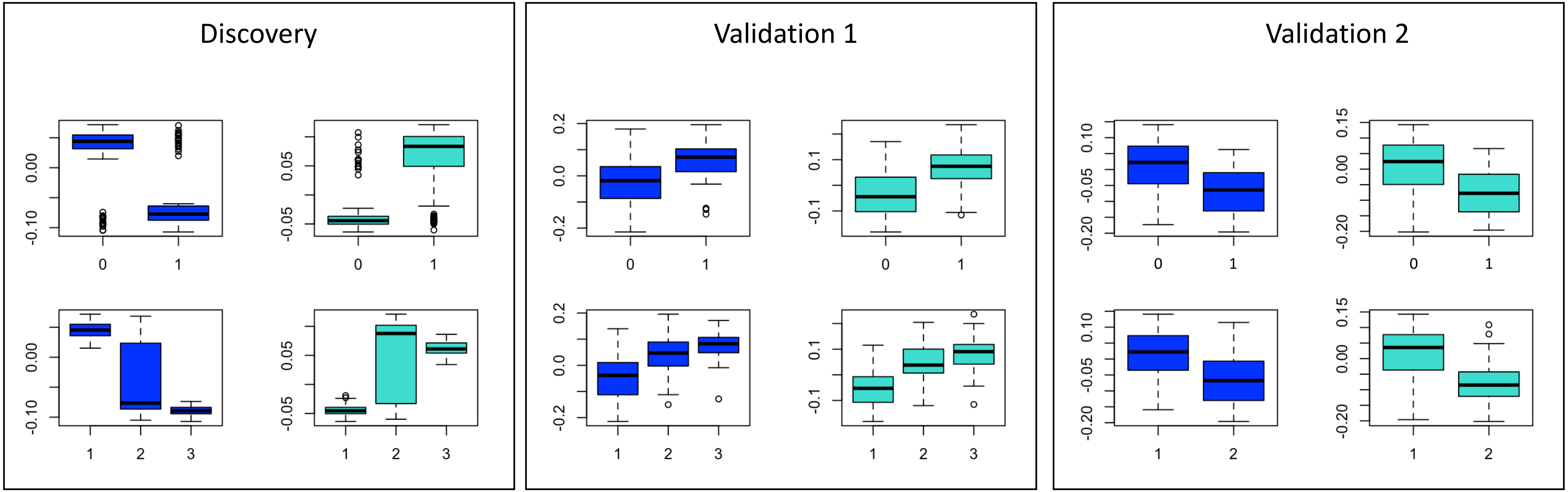

Discovery
Validation 1
Validation 2

**Supplemental Figure 2:** Module metagenes are associated with recurrence and WHO grade in all cohorts. Top row is comparison of metagene scores by recurrence (0 = non-recurrent, 1 = recurrent) and bottom row is comparison of metagene score by WHO grade. We note that all comparisons are significant (t-test $p < 0.05$ for recurrence and ANOVA $p<0.05$ for grade).

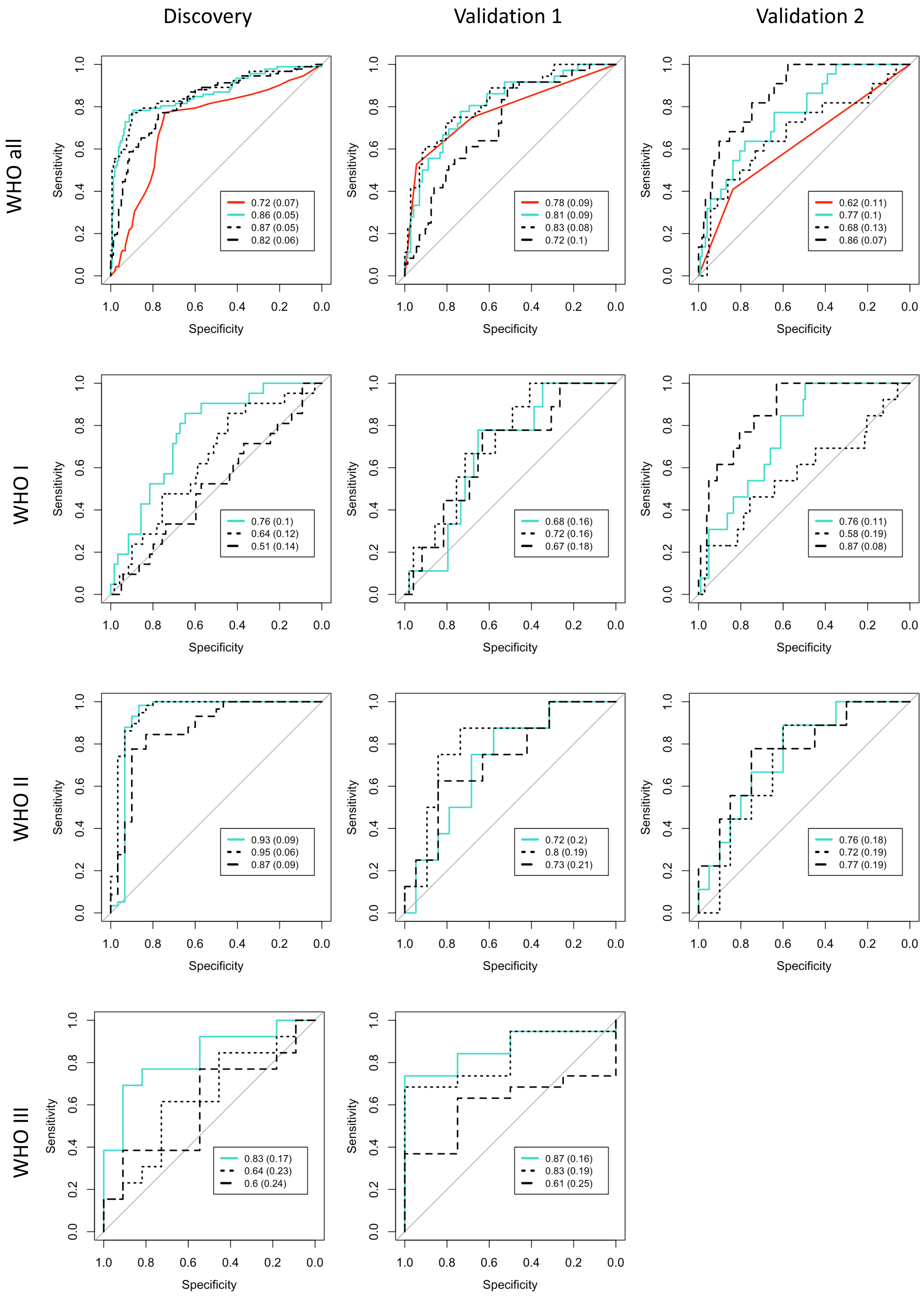
Discovery
Validation 1
Validation 2
WHO all
WHO I
WHO II
WHO III
Sensitivity
Specificity
0.72 (0.07)
0.86 (0.05)
0.87 (0.05)
0.82 (0.06)
0.78 (0.09)
0.81 (0.09)
0.83 (0.08)
0.72 (0.1)
0.62 (0.11)
0.77 (0.1)
0.68 (0.13)
0.86 (0.07)
0.76 (0.1)
0.64 (0.12)
0.51 (0.14)
0.68 (0.16)
0.72 (0.16)
0.67 (0.18)
0.76 (0.11)
0.58 (0.19)
0.87 (0.08)
0.93 (0.09)
0.95 (0.06)
0.87 (0.09)
0.72 (0.2)
0.8 (0.19)
0.73 (0.21)
0.76 (0.18)
0.72 (0.19)
0.77 (0.19)
0.83 (0.17)
0.64 (0.23)
0.6 (0.24)
0.87 (0.16)
0.83 (0.19)
0.61 (0.25)

**Supplemental Figure 3:** Logistic regression classifier performance or sparse modules. For each receiver operative characteristic curve, turquoise represents the full E2F4/FOXM1-enriched module and black represents the sparse models (short dashed = sparse model 1 and long dashed = sparse model 2). The top row (including all WHO grades) includes a red curve which represents the predictive accuracy of WHO grade alone. Note that each model contains a single predictive variable (metagene or WHO grade). Models in rows 2-4 include only individual WHO grades as labeled. Columns (left to right) represent performance on the discovery cohort (10-fold cross validation), merged microarray validation cohort (Validation 1), and RNA-seq validation cohort (Validation 2). We note that neither sparse model exhibits consistently increased accuracy when compared to the full model, which supports the increased generalizability obtained by the redundancy of a larger model.

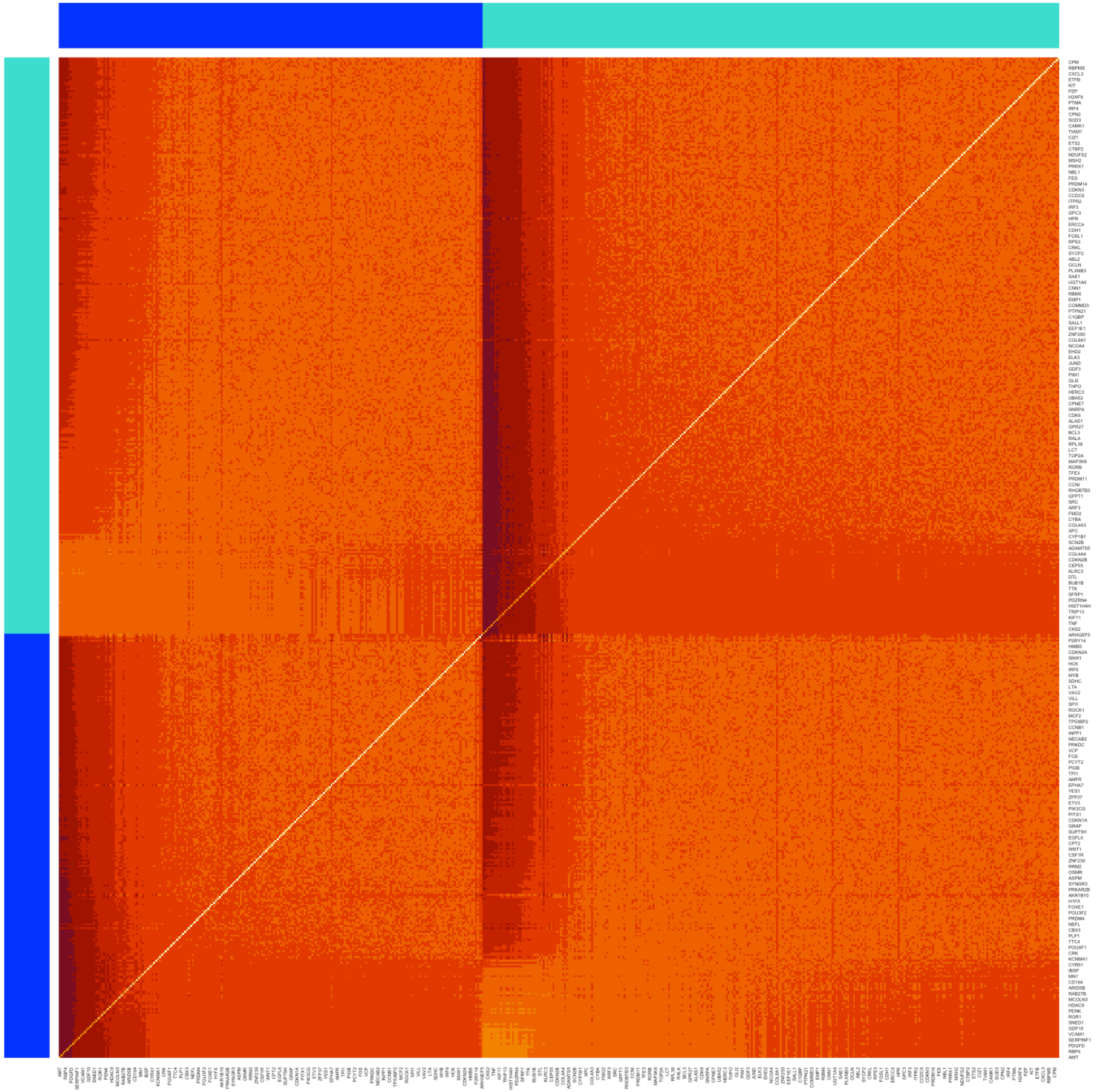

**Supplemental Figure 4:** Heatmap representation of topological overlap map, clustered by module. Diagonal values are set to 0. Darker colours represent higher values.

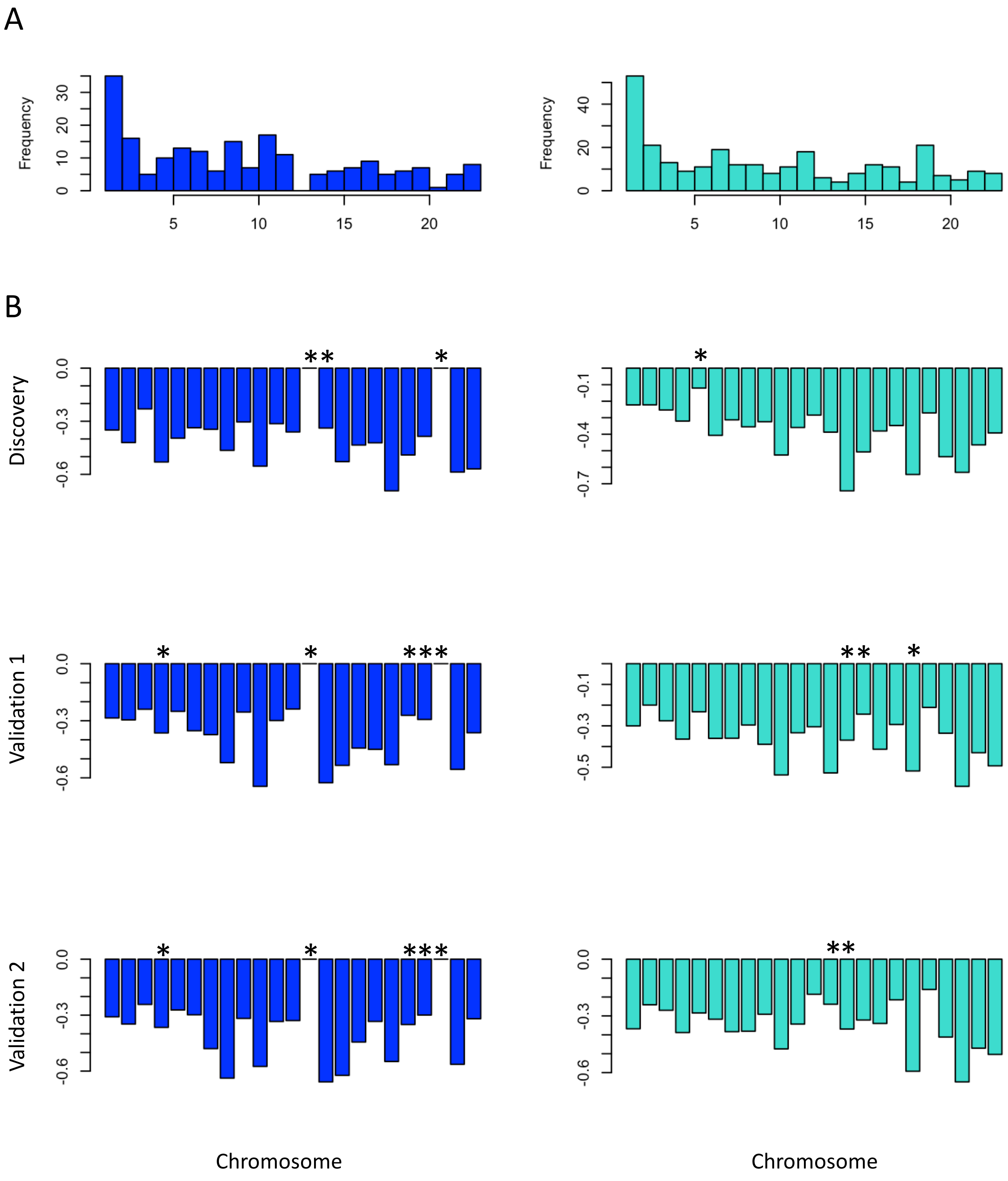

A
Frequency
B
Discovery
Validation 1
Validation 2
Chromosome
Chromosome

**Supplemental Figure 5:** Gene linkage analysis of module genes. A: Histogram representation of module gene loci by chromosome (23 is used to represent the X chromosome). Blue represents the SUZ12-enriched module and turquoise represents the E2F4/FOXM1-enriched module, as in the rest of the paper. We note that these genes are distributed throughout the genome in both modules, and do not reside within a single neighborhood. B: Correlation between gene co-expression and distance on each chromosome. For each pair of modules genes on a chromosome, the difference between their starting position and their co-expression (measured by Pearson correlation) is recorded, then the overall Pearson correlation computed and plotted. Notably, there is a significant negative correlation between gene distance and co-expression throughout the genome, suggesting the importance of regional gains/losses in meningioma biology and providing support to the gene-program-based approach. *Correlation not significant ($p>0.05$).
